# Comparison of Nutrient Intake Across Different Dietary Patterns in Brazilian Community-Dwelling Older Adults

**DOI:** 10.3390/nu17040603

**Published:** 2025-02-07

**Authors:** Hilara Forti Camargo, Agatha Nogueira Previdelli, Angelica Castilho Alonso, Marta Ferreira Bastos, Adriana Machado-Lima, Rita de Cássia de Aquino

**Affiliations:** 1 Postgraduate Program in Aging Sciences, São Judas Tadeu University, São Paulo 05503-001, Brazil; hilara.forti@gmail.com (H.F.C.); angelica.alonso@saojudas.br (A.C.A.); prof.martabastos@usjt.br (M.F.B.); prof.adrianalima@usjt.br (A.M.-L.); 2 Faculty of Health Sciences, Universidad Autónoma de Chile, Providencia 7500912, Chile; agatha.usp@gmail.com

**Keywords:** nutrient intake, dietary pattern, older adult

## Abstract

Background/Objectives: Dietary patterns are able to capture the complexity of the diet. The present study aimed to compare the nutrient intake across different dietary patterns in Brazilian community-dwelling older adults. Methods: Dietary patterns from 295 (predominantly women, 85%) of Brazilian community-dwelling older adults were identified using principal component analysis, based on a 24 h dietary recall (24HR). The following patterns were identified: the “traditional” pattern (consisting of in natura or minimally processed food); the “modified” pattern (consisting of processed foods, confectionery), and the “snack” pattern (composed of coffee, whole milk, bread, toast, butter, or margarine). Participants were divided into three tertiles according to their scores in each pattern. A comparison of energy and nutrient intake across the tertiles of the three patterns was analyzed using the Kruskal–Wallis rank sum test. Then, the intake of energy and macro and micronutrients were analyzed. Results: Older adults with higher scores for the “traditional” pattern had significantly higher intakes of total energy, all macronutrients, beta-carotene, vitamin C, E and K, thiamine, pyridoxine, and total folate (*p* < 0.05). Participants with higher scores for the “modified” pattern had significantly higher intakes of total energy, added sugar, total and animal protein, leucine, arginine, monounsaturated fat, cholesterol, niacin, and pyridoxine; the second tertile had lower vitamin A than the first tertile (*p* < 0.05). Finally, older adults with higher scores for the “snack” pattern had significantly lower intakes of total and insoluble fiber, animal and vegetable proteins, vitamins (A, E, and K), niacin, and pyridoxine (*p* < 0.05). The “Traditional” pattern exhibited the most appropriate dietary pattern regarding the availability of essential nutrients, resulting in a better quality of habitual intake and outcomes that are related to health promotion and reduction in the risk of non-chronic issues. Conclusions: The results reinforce the importance of public policies that encourage the maintenance of the traditional Brazilian dietary pattern, which is in alignment with Brazilian Dietary Guidelines, especially in the older population.

## 1. Introduction

In recent years, diet studies have moved from a traditional approach based on the consumption of nutrients, foods, or food groups, towards a more contemporary methodology, defined as dietary pattern, which is able to capture the complexity of the diet and its effect on health promotion, risk of diseases, population aging, and longevity [1]. The necessity to study the complex interaction between food combinations led nutritional epidemiology to evaluate food habits based on dietary patterns [2].

It is recognized that many factors, such as genetic, physical activity practice, lifestyle, behavior, and socioeconomic status, can affect the life quality of older adults. In this way, the adoption of a healthy dietary pattern is associated with a reduced risk of developing chronic non-communicable diseases (NCDs) and also a better quality of life [3,4,5].

Several dietary patterns are related to the reduced risk of NCDs, such as the Mediterranean diet [6,7,8,9,10,11,12] and MIND diet (Mediterranean-DASH Intervention for Neurodegenerative Delay), whose benefits are associated with brain function. Greater adherence to the MIND diet is associated with lower cognitive decline and greater protection against Alzheimer’s and Parkinson’s disease [13,14,15,16,17]. Although the Brazilian dietary pattern contains health foods such as “rice and bean” it falls short of the quality of MIND and Mediterranean-DASH diets due to the absence of important foods and food groups such as vegetables, fish, and olive oil. Currently, a national Brazilian study identified three dietary patterns among their population: the “traditional pattern”, characterized by a high intake of rice, beans, and meat; the “breads and butter/margarine pattern”, characterized by the high consumption of breads, margarine/butter, and coffee; and the “western pattern”, characterized by a high intake of sodas, pizzas, snacks, flour, and pasta (ultra-processed food) [18].

Recent narrative reviews summarized findings on dietary factors that influence longevity, concluding that healthier dietary patterns tend to have higher nutrient-rich plant foods and a lower prevalence of processed foods. Public health strategies should aim to create healthier food environments so that nutritious options are readily accessible [19,20].

According to Brazilian Dietary Guidelines [21], the main characteristic of a dietary pattern considered as protective is the high amount of in natura foods, including foods of plant origin such as fruits, vegetables, legumes, and whole grains associated with low intake of processed and ultra-processed food. The habitual consumption of certain foods ensures the ingestion of nutrients and bioactive compounds essential to meet dietary recommendations, promoting well-being in old age [22]. There are few studies in the literature that have identified dietary patterns of older people and associated them with nutrient intake. This may allow the identification and understanding of possible nutritional benefits and risks [23,24,25]. It should be highlighted the majority of recent studies among older adults evaluated the dietary intake based on hypothesis-driven approaches, especially dietary indexes, which were developed based on an older version of the Brazilian Dietary Guidelines [26,27]. In this context, the comparison of nutrient intake across different dietary patterns obtained from a data-driven approach could provide additional information that reinforces the importance of the actual Brazilian Dietary Guidelines. Thus, the present study aimed to compare the nutrient intake across different dietary patterns in Brazilian community-dwelling older adults.

## 2. Materials and Methods

### 2.1. Study Design and Participants

This descriptive cross-sectional study was conducted with male and female individuals, aged 60 years and over, attending Integrated Health and Education Centers (IHEC) in São Caetano do Sul, São Paulo, Brazil, which provides assistance to 2000 seniors. Details of sampling methodology and selection were published elsewhere [23]. The data collection was performed from February 2014 to February 2015, in accordance with the Research Ethics Committee.

The sample studied was established by convenience and non-probabilistic criteria. Sample size was determined according to the primary study objective of assessing the dietary intake of older people. Therefore, the method proposed by Hair et al. [25] was used, in which the sample size should be five times larger than the number of items in the Food Frequency Questionnaire (FFQ). The FFQ used in this study consisted of 57 items, so a sample of at least 285 individuals was estimated (considering a 95% confidence level and a sampling error of 5%). Thus, 350 older people were approached to participate in the study and 295 voluntarily agreed to participate. Older adults with cognitive impairment who were unable to answer the interview were excluded.

### 2.2. Data Collection

A team of trained nutritionists conducted the interview in IHEC offices and applied a questionnaire developed to collect sociodemographic, economic, health, and lifestyle data (age, sex, race, marital status, educational level, income, weight and height, diseases, and physical activity).

Food consumption was evaluated using two 24 h recall (24HR), with two-week intervals. The usual intake of energy and nutrients was estimated using the Multiple Source Method (MSM) by removing the intrapersonal variance intake [28]. Moreover, the MSM requires at least a second short-term dietary measurement (such as 24HR) on a random subsample (at least 30%) to produce the usual intake estimates. In the present study, the second 24HR was applied for the overall population. To apply the MSM, the consumption of each 24 HR was assessed in terms of energy (kilocalories), grams, milligrams, or micrograms (depending on the nutrient). For each participant, the usual intake of energy and each nutrient was estimated and adjusted by gender, age, and socioeconomic level. As the MSM was applied only for nutrients, the probabilities of consumption were set as 100%. The interviews were conducted in an attempt to cover all weekdays and months of the year, considering the variability of the food consumption pattern across the days of the week and the four seasons.

Individuals were instructed to write down the food consumed on the day before the interview to reduce the risk of forgetfulness in the collection of 24HR. Food and beverages reported in the 24HR was first criticized (household measurements were converted into standard measurement units). Then, dietary data were entered into the Nutrition Data System for Research (NDS-R) software, version 2013.

### 2.3. Dietary Patterns

The methodology applied to identify the dietary patterns was previously reported by Ferreira et al. [23]. Briefly, principal component analysis (PCA) was performed to obtain dietary patterns, considering food in grams or milliliters. The 529 foods/beverages reported in the 24HR were grouped into 57 predefined food items. A validated FFQ for the Brazilian population was used to define these food items [23]. In the first stage of the PCA, the Cattell graph (scree plot) was evaluated and consumption patterns with eigenvalues higher than 1.0 were identified, which indicates a greater capacity to explain the variance of the data. Then, orthogonal Varimax rotation was used to increase the interpretability of the data, and food items whose factor loading was greater than |0.25| were kept in the matrix. To verify the fitness of this analysis, the uniformity of the data was tested using the Kaiser–Meyer–Olkin test (KMO), adopting values greater than 0.50 as a satisfactory result. The homogeneity of variance was confirmed using Bartlett’s Sphericity test (*p* < 0.001). Factor scores were calculated for each participant for each dietary pattern. The three dietary patterns identified were called the following: the “traditional” pattern (in natura or minimally processed foods—beans, rice, chicken, vegetables, and olive oil); the “modified” pattern (processed foods, confectionery sweets, soft drinks, pasta, industrialized sauce, and pork), and the “snack” pattern (coffee, whole milk with sugar, bread, toast, butter, or margarine). More details of this methodology can be found in a previous publication [23].

To analyze the nutrient intake, participants were divided into three tertiles according to their scores of each pattern. We decided to devise consumption scores into tertiles because it is one of the most widely used and accepted methodology to evaluate dietary pattern analyses. This also helped in interpreting the results. Total energy (kcal), total protein (g) (animal and vegetable), amino acids (leucine and arginine), lipids (saturated, unsaturated, and monounsaturated), cholesterol, linoleic acid, alpha-linolenic acid, omega 3, total carbohydrates, total and added sugar, and fiber (total, soluble, and insoluble) were analyzed. The total supply (mg/mcg) of fat-soluble vitamins (A, beta-carotene, retinol, E, and K); water-soluble vitamins (thiamine, riboflavin, niacin, pantothenic acid, pyridoxine, folate, total, cobalamin, and vitamin C); and mineral intake (calcium, phosphorus, iron, magnesium, zinc, copper, selenium, manganese, sodium, and potassium) were analyzed across the tertiles with adherence to the dietary patterns. The analysis was conducted separately for each pattern.

### 2.4. Statistical Analyses

The study variables were categorized for descriptive and inferential statistical analysis. A Shapiro–Wilk test revealed the absence of normality in the distribution of the data (*p* < 0.05). A Kruskal–Wallis test was performed to compare the energy and macro and micronutrient intake among the tertiles of each pattern. Bonferroni’s post-hoc correction test was applied to multiple comparisons. All statistical analyses were performed using SPSS software for Windows version 22.0 (SPSS Inc. Chicago, IL, USA). The significance level was set at *p* ≤ 0.05 for all analyses.

### 2.5. Ethical Aspects

This study was conducted according to the guidelines in the Declaration of Helsinki, and all procedures involving human subjects were approved by the São Judas University of Ethics Committee under number 470.062, and written informed consent was obtained from all subjects.

## 3. Results

The study participants were 295 older people, 47% of whom were aged between 60 and 69 years, 45% were married, and 62% were retired (Table 1). It is important to highlight that the vast majority of the study population was female (85%). Low level of education was confirmed as 42% of older adults had only a maximum of 4 years of study. Healthy lifestyle habits such as practicing physical activity (87%) and not smoking (96%) were observed in a larger number of participants.

To evaluate the intake of nutrients in the identified dietary patterns, the median of energy, macronutrients (carbohydrates, proteins, and lipids and their fractions), and micronutrients consumption was compared according to the tertiles of each dietary pattern scores (Table 2 and Table 3).

Older adults with higher scores for the “traditional” pattern had significantly higher intakes of total energy and all macronutrients (*p* < 0.05), with the exception of total and added sugar (*p* value was 0.462 and 0.266, respectively) (Table 2). In the second dietary pattern, called “modified”, the participants with higher scores for the “modified” pattern had significantly higher intakes of total energy, added sugar, total and animal protein, leucine, arginine, monounsaturated fat, and cholesterol (*p* < 0.05). Finally, older adults with higher scores for the “snack” pattern had significantly lower intakes of total and insoluble fiber (*p* < 0.05).

The amount of total protein was lower in the higher tertile of the “snack” pattern, as well as the amount of animal and vegetable proteins. The amount of animal protein stood out in the “modified “pattern”, while the consumption of vegetable protein was higher in the “traditional” pattern when compared to the “snack” pattern.

Regarding vitamin consumption, older adults with higher scores for the “traditional” pattern had significantly higher intakes of beta-carotene, vitamin C, E and K, thiamine, pyridoxine, and total folate (*p* < 0.05) (Table 3). Participants with higher scores for the “modified” pattern had significantly higher intakes of niacin and pyridoxine (*p* < 0.05), the second tertile has lower vitamin A than the first tertile (T1). Older adults with higher scores for the “snack” pattern had significantly lower intakes of vitamin A, E and K, niacin, and pyridoxine (*p* < 0.05).

Finally, for the relation to minerals, older adults with higher scores for the “traditional” pattern had significantly higher intakes of all minerals (*p* < 0.05), with the exception of calcium where the *p* value indicated no significant differences (*p* > 0.05) (Table 3). Participants with higher scores for the “modified” pattern had significantly higher intakes of phosphorus, iron, zinc, and sodium (*p* < 0.05). Older adults with higher scores for the “snack” pattern had significantly lower intakes of phosphorus, iron, and selenium (*p* < 0.05).

Calcium and vitamin D are particularly important for older adults, especially among the female population. However, no statistically significant differences (*p* < 0.05) were found between the dietary pattern tertiles. The low intake of these nutrients resulted in a higher prevalence of inadequate intake of vitamin D in the three patterns (“traditional”, “modified”, and “snack”, which were 96%, 98%, and 99%, respectively). For calcium, the prevalence was 81%, 79% and 86%, respectively.

## 4. Discussion

This study evaluated the dietary patterns and found important nutritional differences, which can affect the diet quality of Brazilian community-dwelling older adults. The dietary patterns identified were the “traditional” pattern, composed of in natura foods that are much consumed by the population (beans, rice, chicken, and vegetables); the “modified” pattern”, consisting of processed foods (confectionery sweets, soft drinks, pasta, industrialized sauces, and pork), foods that are easy to purchase and prepare; and the “snack” pattern, composed of foods usually used for breakfast or in intermediate meals, which are also widely used in the replacement of meals, especially dinner (coffee, whole milk with sugar, bread, toast, butter, or margarine).

The “Traditional” pattern, also called the “healthy” pattern by the literature, was somewhat similar to the “Mediterranean” pattern, as both are rich in vegetables and a source of several vitamins, minerals, and fibers. It should be noted that the consumption of “rice and beans” (in the same pattern) provided adequate amounts of all the essential amino acids. Furthermore, the consumption of chicken-provided protein with high biological value is essential to highlight. The “traditional “pattern found in the present study was better than the national study, since it does not have red meat, which is associated with an increased risk for NCD [18]. A scoping review with six observational studies investigated the association between dietary patterns, osteoporosis, and fracture risk [29]. The study concluded that a Mediterranean diet in non-Mediterranean populations may reduce the risk of hip fracture, and a DASH (Dietary Approaches to Stop Hypertension) may improve bone mineral density.

Otherwise, the “modified” and “snack” patterns are like “Western” patterns, characterized by a greater presence of processed and ultra-processed foods. These foods are dense in high energy, rich in saturated fat, trans fat, added sugar, and sodium; they have low nutritional density, most of them are poor in fibers and micronutrients; and they can also contain many additives.

In a recent systematic review [30], the consumption of ultra-processed foods was associated with obesity, hypertension, and metabolic syndrome. The classification defined as “NOVA” stands out in nutritional epidemiology when evaluating the effects of food processing level on health outcomes. The word “NOVA” is a name of a classification system in which foods are categorized according to their processing levels. The results of the review by Santos et al. [31] suggested that the consumption of ultra-processed foods may be associated with a range of adverse health outcomes, especially in the aging population.

One very important nutrient for older adults is protein. The PROT-AGE (PROT-AGE Study Group) recommends higher protein consumption for individuals over 65 years to maintain muscle mass [32]. In the present study, the upper tertile of the “snack” pattern presented lower quantities of protein. The “modified” and “traditional” patterns found the opposite result. The “snack” pattern had whole milk as the main source of animal protein, and the latte snack probably replaced another meal that could contain several other sources of protein. Meeting protein recommendations is associated with a better overall diet quality, which can protect lean mass. So, assessing the individual characteristics that may affect protein intake is crucial to help older people meet their protein needs [33].

Results found by Gaspareto et al. [34], who evaluated protein consumption in the same group of individuals, found that at lunch, meals composed of meat, cereals, and legumes contributed a greater amount of total protein, as observed in the “traditional” pattern. Besides that, they concluded that older people who tended to replace dinner for a poor meal had a reduced protein intake, as observed in the dietary pattern called “Snack”. Another important finding showed that 50% of these older people presented protein intake below the amount recommended by the PROT-AGE (1.0 g to 1.2 g/kg/day). Protein consumption ensures the intake of essential amino acids, including leucine and arginine. Older adults with higher scores for the “traditional” pattern had a significantly higher intake of total protein, which can positively affect the healing and tissue regeneration process due to high arginine intake, as well as impact the recovering of muscle mass that can result in lower fragility, owing to high leucine consumption [35].

Dietary fibers presented differences between the “traditional” and “snack” patterns. A higher amount of total and insoluble fibers was observed in the upper tertile of the “traditional” pattern, which contains food sources such as beans and vegetables. On the other hand, older adults with higher scores for the “snack” pattern had significantly lower intakes of total and insoluble fiber (*p* < 0.05). According to the Institute of Medicine (IOM) [36], dietary fibers have diverse functions, such as promoting satiety, which can contribute to reducing energy intake and therefore reduce the risk of obesity and increase blood glucose and serum lipoprotein levels. In addition, the consumption of food sources of dietary fiber favors the availability of bioactive and phytochemical compounds with beneficial functions, such as antioxidant and anti-inflammatory properties [37,38].

The antioxidant activity of some nutrients such as beta-carotene can have an impact on the effects of free radicals and consequently on cognitive protection [39,40,41]. The results found that the upper tertile of the “traditional” pattern presented higher, possibly due to the presence of vegetables. No statistical differences were found for the other patterns (*p* > 0.05). Another nutrient with important antioxidant properties whose intake is reduced in older individuals is vitamin E. A study that investigated the dietary intake of antioxidants (nutrients and non-nutrients) in a Spanish older population (mean aged 80 years), observed an adequate intake of antioxidant vitamins such as vitamin A and vitamin E. It suggested that the Mediterranean pattern, composed of fruits, vegetables, and olive oil, provided greater amounts of antioxidant substances [42]. Based on these data, it is important to evaluate the intake of vitamin E in the older population, since this vitamin has important functions in oxidative stress response, immune function, and cardiovascular and nervous systems [43]. As shown in the results, the higher tertile of the “traditional” patterns had a higher intake of vitamin E.

Minerals with the highest consumption in the higher “traditional” pattern tertile were magnesium, iron, zinc, copper, selenium, manganese, sodium, and potassium. Magnesium is the fourth most abundant mineral in the body and is recognized as a co-factor for more than 300 enzymatic reactions [44]. Magnesium is necessary for protein synthesis and regulation of muscle contraction, blood pressure, cardiac excitability, nerve transmission, and neuromuscular conduction. Its role in the prevention and treatment of neurological diseases has also been studied [45]. It was observed in our study that magnesium consumption did not show statistically significant differences between the “modified” and “snack” pattern tertiles. Zhang et al. [46] explored the association between dietary pattern and cognitive function in the Chinese population. They found a dietary pattern called “vegetable-pork” (characterized by a higher intake of legumes, vegetables, fruits, nuts, pork, fish, and plant oil). This pattern, with a higher amount of vitamin C and E, zinc, iron, copper, and selenium, was associated with better cognitive function in older adults.

Older adults with higher scores for the “traditional” pattern had significantly higher intakes of iron and folate, probably due to the presence of vegetables and meat. But older adults with higher scores for the “snack” pattern did not have significantly higher intakes of these nutrients, despite observing a greater consumption of breads, which contains enriched flour, iron, and folate. An inadequate intake of iron and folate is one of the risk factors for anemia, which is very common in older adults and associated with an increased risk of physical, functional and cognitive impairment, hospitalization, and mortality [46,47,48,49].

In this study, copper consumption was higher in the upper tertile of the “traditional” pattern, which may be related to a lack of food sources such as legumes. Copper acts as an enzymatic co-factor ensuring the catalytic property of several antioxidant enzymes, in which deficiency may be related to cognitive impairment, as well as several essential micronutrients such as zinc and selenium, which are involved in protein coding, genomic stability, and telomere integrity [50,51]. For the other dietary patterns, there was no statistical significance in copper consumption (*p* > 0.05). Bagheri et al. [24] studied the relationship between dietary pattern, nutrient intake, and sarcopenia, and the pattern rich in unsaturated fat, copper, vitamin E, magnesium, and iron showed a lower frequency of sarcopenia.

Dietary patterns have been changed in most countries and the main changes involve the replacement of in natura foods and culinary preparations by industrialized products ready for consumption (processed and ultra-processed food). These transformations mainly determine an imbalance in the supply of nutrients, excessive energy intake, and low dietary diversity, which are associated with cognitive disorders and physical function impairment [52,53,54]. Results from this current study reinforce the importance of developing public health promotion policies that focus on the following: (1) increasing accessibility to healthy foods; (2) improving the supply of care; and (3) providing adequate assistance facilities for older adults. It is important to call attention to the fact the “Traditional” pattern was associated with males and the practice of physical activity (*p* < 0.05), the “Modified” pattern was also associated with the male gender and retired individuals (*p* < 0.05), and the “snack” pattern was associated with an age of 80 years or older (*p* < 0.05) [23]. However, despite these differences, public policies are usually developed to the overall population and particularities such as age, gender, and/or socioeconomic status are not taken into account. This includes the Dietary Guideline for Brazilian population that was developed for children over 2 years old. Encouraging the consumption of fresh food and reducing the intake of processed and ultra-processed foods can be done through several measures including the following: (1) food label information (emphasizing the amount of fats, salt, and sugar in industrialized products), (2) tax incentives (healthy food subsidies and unhealthy food taxation), and (3) educational campaigns.

This study presents some limitations that should be considered. First, the cross-sectional design has inherent limitations in establishing a habitual diet; however, consumption was adjusted using the Multiple Source Method (MSN) to remove intrapersonal variance. Second, the study population was composed predominantly of women, which could impact the identification of overall dietary patterns. However, the higher proportion of women was expected since, in the study city, female gender represents more than 60% of the older adult population [55]. Also, epidemiological studies usually estimate the dietary patterns for overall population, rather than subgroups, using gender, socioeconomic, and demographic characteristics to adjust the results. Finally, the identification of data-driven dietary patterns is more appropriate in females than males [56]. However, as previously stated, the majority of recent studies among older adults evaluated the dietary intake based on hypothesis-driven approach, especially dietary indexes [26,27], so the data-driven approach used in the current research is a differential of the present study. Another possible limitation is the collection period that occurred 10 years ago. The acquisition and availability of food may have changed along the years, impacting the dietary patterns. However, the Brazilian dietary patterns reported by one of the most recent publications were very similar to those found in the present study [18]. Another possible limitation is age, as common changes in aging, such as difficulty concentrating and decreased memory, can increase the duration of the interview and require greater attention from the interviewer. As age can also have a negative impact on recording the information about food intake, the use of 24HR can be a limitation. In the present study, all participants wrote down all food and beverages consumed on the day before the interview to reduce the risk of forgetfulness in the collection of 24HR. This procedure was carried out on both days of collection. In this sense, the training of the nutritionists engaged in the research and the space available for the collection were essential to positively influence the reliability of the information collected and used in the analysis and results. Furthermore, older people may have an influence on their dietary patterns if they are dependent on the acquisition and preparation of food. Consequently, the findings may not be generalizable and this warrants further improvement in study designs with older adults. Finally, the potential for selection bias due to the use of a non-probabilistic sampling method can hinder the comparison between national and international population studies. To minimize this effect, all participants were selected from fifteen neighborhoods geographically distributed in the city, in order to capture differences in sociodemographic and economic factors.

## 5. Conclusions

In conclusion, higher adherence to the “traditional” pattern, composed of in natura or minimally processed foods (beans, rice, chicken, vegetables, and olive oil), resulted in higher healthy nutrient intakes, such as leucine, arginine, fiber, vitamins, and minerals. This result demonstrates that the nutritional quality of each pattern and food choices can impact the healthy status of older people, as food choices are associated with the availability of nutrients and bioactive compounds. In this context, identifying the dietary pattern of older people and relating it to the intake of nutrients makes it possible to outline appropriate nutritional strategies to promote the health and quality of life of older people.

Another important point to highlight is that this is the first study that evaluated dietary patterns in older adults living in a city with one of the highest HDI (Human Development Index) in Brazil. As the HDI is a socioeconomic indicator that evaluates the development of a population based on three dimensions (income, education, and health), a better dietary quality and better nutrient intake profile were expected. However, the patterns identified were in agreement with those found in other Brazilian surveys. So, the results reinforce the importance of public policies that encourage the maintenance of the traditional Brazilian pattern that is in alignment with actual Brazilian Dietary Guidelines, which suggest eating less processed food and avoiding ultra-processed food. In other words, they reinforce the importance of consuming healthy and traditional Brazilian foods such as rice, beans, greens, legumes, and fruits while encouraging a reduction in the intake of candies, pasta, margarine, soft drinks, and sugar.

Future studies are important to investigate how dietary patterns impact nutrient intake and the recommendations of local dietary guidelines.

## Figures and Tables

**Table 1:** Sociodemographic and lifestyle characteristics of older adults living in municipality of São Caetano do Sul, SP, Brazil, 2015.

Variables	*n*	%
Gender		
Female	251	85.1
Male	44	14.9
Age (years)		
60–69	137	46.5
70–79	124	42
80 or older	34	11.5
Marital Status		
Married	132	44.7
Widowed	107	36.3
Separated/Divorced	56	19
Lives alone		
Yes	75	25.4
No	220	74.6
Schooling (years of study)		
0–4 years	123	41.7
5–8 years	62	21
9–12 years	53	18
12 years or more	57	19.3
Employment status		
Works	21	7.1
Retired	184	62.4
Homemaker	86	29.2
Physical activity		
Yes	256	86.8
No	39	13.2
Smoking habit		
Yes	13	4.4
No	282	95.6

**Table 2:** Energy and macronutrient distribution across dietary pattern tertiles, São Caetano do Sul, Brazil, 2015.

Energy and Nutrients	Dietary Pattern											
	“Traditional”				“Modified”				“Snack”			
	T1	T2	T3	*p* Value	T1	T2	T3	*p* Value	T1	T2	T3	*p* Value
Energy (kcal)	1354.26	1412.88	1706.45	**<0.001 ^b,c^**	1484.76	1363	1568.12	**0.001 ^b^**	1479.65	1469.27	1517.4	0.717
Carbohydrate (g)	181.84	183.39	216.11	**<0.001 ^b,c^**	198.44	179.39	201.21	0.051	193.29	183.52	199.73	0.506
Total Sugar (g)	80.58	81.82	80.66	0.462	80.66	77.99	85.23	0.068	81.89	78.72	84.86	0.452
Added sugar (g)	31.34	25.34	28.14	0.266	31.34	22.77	31.74	**0.015 ^a,c^**	27.38	26.14	34.57	0.051
Total fiber (g)	15.41	16.56	20.34	**<0.001 ^b,c^**	17.67	17.34	17.86	0.271	18.83	17.62	16.72	**0.035 ^b^**
Soluble fiber (g)	4.87	4.84	5.78	**0.035 ^b^**	5.24	4.96	5.14	0.595	5.49	5.06	4.95	0.383
Insoluble fiber (g)	10.79	11.3	14.4	**<0.001 ^b,c^**	11.74	12.03	12.65	0.215	13.18	12.13	11.13	**0.006 ^b^**
Total Protein (g)	61.01	66.05	77.56	**<0.001 ^b,c^**	61.31	63.69	77.02	**<0.001 ^b,c^**	70.26	66.63	64.84	0.12
Animal Protein (g)	42.62	46.52	50.09	**<0.001 ^b^**	41.37	43.98	55.74	**<0.001 ^b,c^**	47.09	47.3	44.98	0.116
Vegetable Protein (g)	18.52	18.88	23.76	**<0.001 ^b,c^**	21.16	19.01	21.39	0.107	21.07	20.05	21.04	0.998
Total Leucine (g)	4.63	5.12	5.98	**<0.001 ^b,c^**	4.62	4.95	5.92	**<0.001 ^b,c^**	5.49	5.15	5.14	0.113
Arginine (g)	3.18	3.56	4.16	**<0.001 ^b,c^**	3.34	3.49	4.12	**<0.001 ^b,c^**	3.79	3.59	3.54	0.099
Fat (g)	47.49	48.37	56.65	**<0.001 ^b,c^**	50.69	46.96	53.39	**0.003 ^b^**	49.04	48.49	53.18	0.459
Saturated fat (g)	15.31	15.16	18.21	**0.002 ^b,c^**	16.31	15.13	17.56	**0.024 ^b^**	15.71	15.95	17.32	0.109
Monounsaturated fat (g)	17.1	16.87	20.52	**<0.001 ^b,c^**	18.61	16.19	19.08	**0.002 ^b,c^**	17.73	18.07	19.24	0.583
Unsaturated fat (g)	10.31	10.65	12.59	**<0.001 ^b,c^**	11.69	10.45	11.68	0.053	11.66	10.83	11.14	0.417
Cholesterol (g)	178.34	183.28	197.27	**0.011 ^b^**	182.35	178.92	201.1	**<0.001 ^b,c^**	194.42	184.77	184.64	0.475
Linoleic acid (g)	8.88	8.91	10.66	**<0.001 ^b,c^**	9.99	8.77	9.72	0.055	9.69	9.28	9.56	0.857
Alpha-linolenic acid (g)	1.19	1.29	1.48	**<0.001 ^b,c^**	1.38	1.3	1.32	0.622	1.4	1.29	1.33	0.204
Omega 3 (g)	1.26	1.38	1.59	**<0.001 ^b,c^**	1.46	1.38	1.44	0.42	1.52	1.35	1.38	0.056

Kruskal–Wallis test—Tertile 1 (T1), Tertile 2 (T2), and Tertile 3 (T3). ^a^: T1 ≠ T2 (*p* < 0.05), ^b^: T1 ≠ T3 (*p* < 0.05), ^c^: T2 ≠ T3 (*p* < 0.05).

**Table 3:** Micronutrient distribution across dietary pattern tertiles, São Caetano do Sul, Brazil, 2015.

Vitamins and Minerals	Dietary Pattern											
	“Traditional”				“Modified”				“Snack”			
	T1	T2	T3	*p* Value	T1	T2	T3	*p* Value	T1	T2	T3	*p* Value
Vitamins												
Vitamin A (µg)	617.26	679.01	745.44	0.14	696.41	607.95	692.43	**0.033 ^a^**	712.36	669.77	625.88	0.033 ^a^
Beta-carotene (µg)	3213.36	3874.19	5332.06	**0.001 ^b^**	4655.4	3630.13	4162.39	0.167	4642.04	4365.79	3277.31	0.167
Retinol (µg)	304.04	292.25	279.17	0.672	310.26	270.4	298.33	0.137	292.46	270.91	320.67	0.137
Vitamin D (µg)	3.56	3.88	4.08	0.515	4.14	4	3.55	0.29	3.7	3.56	4.74	0.29
Vitamin E (mg)	4.43	4.79	5.88	**<0.001 ^b,c^**	5.32	4.45	5.3	**0.002 ^a,c^**	5.42	5.06	4.79	0.002 ^a,c^
Vitamin K (µg)	125.59	143.9	165.64	**<0.001 ^d^**	150.47	134.25	150.19	**0.028 ^a^**	156.33	143.3	138.7	0.028 ^a^
Thiamine (mg)	1.16	1.22	1.34	**<0.001 ^b,c^**	1.25	1.16	1.26	0.124	1.25	1.17	1.26	0.124
Riboflavin (mg)	1.4	1.47	1.5	0.261	1.46	1.43	1.52	0.086	1.49	1.41	1.49	0.086
Niacin (mg)	14.89	15.71	18.45	**<0.001 ^b,c^**	15.42	15.27	18.44	**<0.001 ^b,c^**	16.86	15.93	15.79	**<0.001 ^b,c^**
Pantothenic acid (mg)	4.18	4.21	5.19	**<0.001 ^b,c^**	4.59	4.28	4.76	0.029 ^c^	4.84	4.37	4.48	**0.029 ^c^**
Pyridoxine (mg)	1.39	1.48	1.64	**<0.001 ^b,c^**	1.43	1.5	1.63	**0.001 ^b,c^**	1.64	1.47	1.49	**0.001 ^b,c^**
Total folate (µg)	284.09	301.19	355.34	**<0.001 ^b,c^**	320.14	287.57	315.92	0.017	318.19	300.96	313	**0.017 ^a,c^**
Cobalamin (µg)	2.96	3.09	3.15	0.431	3.04	2.89	3.19	0.621	3.16	2.96	3.09	0.621
Minerals												
Vitamin C (mg)	121.58	149.55	154.93	**0.010 ^b,c^**	121.58	143.47	155.28	0.311	173.68	142.41	117.53	0.311
Calcium (mg)	680.2	750.52	764.31	0.202	749.29	715.82	726.91	0.576	753.94	705.79	735.17	0.576
Magnesium (mg)	210.24	216.97	258.37	**<0.001 ^b,c^**	220.24	218.02	235.17	0.051	241.97	220.6	215.44	0.056
Iron (mg)	8.65	9.22	11.7	**<0.001 ^b,c^**	9.83	9.03	10.87	0.002 c	10.04	9.56	9.9	**0.002 ^b^**
Zinc (mg)	7.87	8.51	10.3	**<0.001 ^b,c^**	8.48	8.33	9.39	**0.015 ^b,c^**	8.74	8.77	8.47	**0.015 ^b,c^**
Copper (µg)	847.15	900.83	1078.11	**<0.001 ^b,c^**	908.97	908.81	979.48	0.056	988.66	922.03	914.56	0.056
Selenium (µg)	87.18	91.05	104.37	**<0.001 ^b,c^**	89.72	89.61	101.1	0.009 c	97.69	94.22	92.65	**0.009 ^c^**
Manganese (mg)	2.14	2.42	2.93	**<0.001 ^b,c^**	2.62	2.55	2.54	0.514	2.68	2.58	2.41	0.514
Sodium (mg)	2353	2251.78	2958.55	**<0.001 ^b,c^**	2515.9	2333.25	2649.01	**0.006 ^c^**	2483.3	2543.64	2481.14	**0.006 ^c^**
Potassium (mg)	2152.63	2235.56	2550.94	**<0.001 ^b,c^**	2195.92	2310.79	2462.69	0.053	2525.73	2265.72	2265.59	0.053

Kruskal–Wallis test—differences between tertiles. ^a^: Tertile 1 vs. Tertile 2, *p* < 0.05; ^b^: Tertile 1 vs. Tertile 3, *p* < 0.05; ^c^: Tertile 2 vs. Tertile 3, *p* < 0.05; ^d^: Tertile 1 vs. Tertile 2 vs. Tertile 3, *p* < 0.05.

## Data Availability

The data presented in this study are available on request from the corresponding author due to ethical reasons.
